# Investigating Brain‐Kidney Connection: Effect on Cerebral Blood Flow with Different Levels of Estimated Glomerular Filtration Rate in Normal Brains

**DOI:** 10.1002/alz70856_102712

**Published:** 2025-12-24

**Authors:** Hak Young Rhee, Geon‐Ho Jahng, Jin Yong Lee

**Affiliations:** ^1^ Kyung Hee University Hospital at Gangdong, Seoul, Korea, Republic of (South)

## Abstract

**Background:**

Cerebral blood flow (CBF) plays a pivotal role in maintaining brain function and metabolism, serving as a crucial parameter for assessing brain health. Similarly, the estimated glomerular filtration rate (eGFR) measures kidney function and determines kidney disease stages. CBF and eGFR assess the vascular health of two essential organs—the brain and the kidney.

**Method:**

We enrolled 785 healthy middle‐aged and older adults undergoing regular health check‐ups at our Health Promotion Center. Participants underwent blood tests for creatinine, cholesterol, and diabetes markers, and brain MRIs using a 3T system with pseudo‐continuous arterial spin labeling (pCASL) to measure CBF. Based on their eGFR, calculated from creatinine levels, participants were classified into three stages. We conducted voxel‐based and region‐of‐interest analyses to compare CBF as well as brain tissue volumes across stages and to examine their associations with eGFR and creatinine levels.

**Result:**

The study comprised 368 participants in Stage 1 (high eGFR), 287 in Stage 2 (moderate eGFR), and 130 in Stage 3 (low eGFR). Significantly higher CBF was observed in Stages 1 and 2 compared to Stage 3, notably in the hippocampus and parahippocampal gyrus. CBF showed a positive correlation with eGFR levels and a negative correlation with creatinine levels across all defined brain areas.

**Conclusion:**

CBF was sensitive to differences between eGFR stages, higher in Stage 1 compared to Stages 2 and 3, suggesting its utility as an early marker for brain integrity changes. Its positive correlation with eGFR and inverse relation with creatinine support its role in monitoring early cerebral perfusion changes linked to renal function. This study underscores the interconnectedness of renal and brain health, proposing CBF as a potential early biomarker for cerebral changes in a healthy population.

